# Dietary *Moutan Cortex Radicis* Improves Serum Antioxidant Capacity and Intestinal Immunity and Alters Colonic Microbiota in Weaned Piglets

**DOI:** 10.3389/fnut.2021.679129

**Published:** 2021-06-17

**Authors:** Miaomiao Bai, Hongnan Liu, Shanshan Wang, Qingyan Shu, Kang Xu, Jian Zhou, Xia Xiong, Ruilin Huang, Jinping Deng, Yulong Yin, Zheng'an Liu

**Affiliations:** ^1^Hunan Provincial Key Laboratory of Animal Nutritional Physiology and Metabolic Process; National Engineering Laboratory for Pollution Control and Waste Utilization in Livestock and Poultry Production; Key Laboratory of Agro-ecological Processes in Subtropical Region; Hunan Provincial Engineering Research Center for Healthy Livestock and Poultry Production; Scientific Observing and Experimental Station of Animal Nutrition and Feed Science in South-Central, Ministry of Agriculture, Institute of Subtropical Agriculture, Chinese Academy of Sciences, Changsha, China; ^2^College of Animal Science, South China Agricultural University, Guangzhou, China; ^3^Key Laboratory of Plant Resources/Beijing Botanical Garden, Institute of Botany, Chinese Academy of Sciences, Beijing, China

**Keywords:** *Moutan cortex radicis*, antioxidant capacity, intestinal microbiota, NF-κB signaling, weaned piglets

## Abstract

**Background:** *Moutan cortex radicis* (MCR), as a common traditional Chinese medicine, has been widely used as an antipyretic, antiseptic, and anti-inflammatory agent in China.

**Objectives:** This study aimed to investigate the effects of dietary MCR supplementation on the antioxidant capacity and intestinal health of the pigs and to explore whether MCR exerts positive effects on intestinal health *via* regulating nuclear factor kappa-B (NF-κB) signaling pathway and intestinal microbiota.

**Methods:** MCR powder was identified by LC-MS analysis. Selected 32 weaned piglets (21 d of age, 6.37 ± 0.10 kg average BW) were assigned (8 pens/diet, 1 pig/pen) to 4 groups and fed with a corn-soybean basal diet supplemented with 0, 2,000, 4,000, and 8,000 mg/kg MCR for 21 d. After the piglets were sacrificed, antioxidant indices, histomorphology examination, and inflammatory signaling pathway expression were assessed. The 16s RNA sequencing was used to analyze the effects of MCR on the intestinal microbiota structure of piglets.

**Results:** Supplemental 4,000 mg/kg MCR significantly increased (*P* < 0.05) the average daily weight gain (ADG), average daily feed intake (ADFI), total antioxidative capability, colonic short-chain fatty acids (SCFA) concentrations, and the crypt depth in the jejunum but decreased (*P* < 0.05) the mRNA expression levels of interferon γ, tumor necrosis factor-α, interleukin-1β, inhibiting kappa-B kinase β (IKKβ), inhibiting nuclear factor kappa-B (IκBα), and NF-κB in the jejunum and ileum. Microbiota sequencing identified that MCR supplementation significantly increased the microbial richness indices (Chao1, ACE, and observed species, *P* < 0.05) and the relative abundances of *Firmicutes* and *Lactobacillus* (*P* < 0.05), decreased the relative abundances of *Bacteroides, Parabacteroides, unidentified_Lachnospiraceae*, and *Enterococcus* (*P* < 0.05) and had no significant effects on the diversity indices (Shannon and Simpson, *P* > 0.05). Microbial metabolic phenotypes analysis also showed that the richness of aerobic bacteria and facultative anaerobic bacteria, oxidative stress tolerance, and biofilm forming were significantly increased (*P* < 0.05), and the richness of anaerobic bacteria and pathogenic potential of gut microbiota were reduced (*P* < 0.05) by MCR treatment. Regression analysis showed that the optimal MCR supplemental level for growth performance, serum antioxidant capacity, and intestinal health of weaned piglets was 3,420 ~ 4,237 mg/kg.

**Conclusions:** MCR supplementation improved growth performance and serum antioxidant capacity, and alleviated intestinal inflammation by inhibiting the IKKβ/IκBα/NF-κB signaling pathway and affecting intestinal microbiota in weaned piglets.

## Introduction

Oxidative stress often causes the damage of mammalian tissue cells, especially the intestine, which significantly affects the health status and decreases the performance (1, 2). Various challenges, such as changes in feed nutrition and environment and pathogenic micro-organisms, as well as vaccine and drugs use, induce oxidative stress for weaning piglets (3). Especially, weaning stress causes a decrease in food intake, impaired intestinal barrier function, and disordered intestinal microbiota, which further leads to the damage of immune function and increase of the susceptibility to disease (4, 5). Antioxidative enzymes forming an antioxidative defense system protect the body against reactive oxygen species (ROS) overproduction. Besides, oxidative stress and inflammation are closely related. Cytokines are activated and secreted when the systemic inflammatory incidences. The activation of nuclear factor kappa-B (NF-κB), a transcription factor, can promote the expression of pro-inflammatory cytokines (6). Antibiotics have been used as growth promoters and immune enhancers at subtherapeutic levels in feed for many years. However, in recent years, the reduction or removal of dietary antibiotics has become a developing tendency in swine production (7). Therefore, finding effective and safe feed additives as alternatives to antibiotics is a strong demand for the swine industry. Traditional Chinese medicine is a natural substance, safe, and reliable with little toxicity (8). Due to the extensive antibacterial and synergistic effects, traditional Chinese medicine has no drug resistance and overcomes the shortcomings of antibiotics.

*Moutan cortex radicis* (MCR) from the tree peony (*Paeonia suffruticosa*), commonly known as “Mu Dan Pi,” is a traditional Chinese medicine commonly used for anti-inflammatory, analgesic, antispasmodic, and anti-oxidation properties (9, 10). Traditionally, MCR has the functions of clearing heat, promoting blood circulation and removing blood stasis, and alleviating human diseases. Previous studies have demonstrated that MCR had potent free radicals and superoxide anion radicals scavenging capacity and inhibited ROS production for alleviating oxidative stress (11, 12). MCR is rich in various chemical components, including paeonol, paeoniflorin, oxypaeoniflorin, galloylpaeoniflorin, and gallic acid (13). Paeonol is known to be the main active ingredient, which is reported to inhibit blood coagulation and platelet aggregation for enhancing blood circulation (14) and reduce the production of pro-inflammatory cytokines (15). Moreover, MCR and its bioactive components have also been reported to alleviate obesity, diabetes, and inflammation (16, 17). Previous studies showed that in neuro-inflammatory therapy, paeonol inhibited IκBα to suppress the translocation of NF-κB and decrease the release of pro-inflammatory products (18). In addition, the gut microbiota is the important contributor to animal health and growth such as nutritional conversion, immunity, and intestinal mucosal barrier function (19). MCR is usually used in formulating traditional Chinese medicine additives to contribute to preventing diarrhea and increasing liveweight gain of fattening pig. However, the positive effects of MCR on weaning stress, inflammatory response, and the gut microbiota composition of weaned piglets have not been reported. In the present study, the effects of dietary MCR on growth performance, serum antioxidant indexes, intestinal morphology, anti-inflammatory response, and the gut microbiota composition in weaned piglets were explored.

## Materials and Methods

### Plant Material and LC–MS Analysis

*Moutan cortex radicis* was provided by the Institute of Botany, Chinese Academy of Sciences (Beijing, China). Fresh MCR was carefully cleaned and dried for about 1 week at 37°C, then completely crushed using a pulverizer, and passed through a 60-mesh sieve. A 20 g of powder samples were extracted with 100 mL 75% ethanol (V:V) in a conical bottle, mixed, and sonicated in an ultrasonic instrument (300 W, 50 kHz, 30°C) for 1 h. The mixture was filtered and the filtrate was diluted, passed through a 0.22-μm filter membrane, for it to be then used directly for LC–MS analysis.

The mobile phase system of LC–MS for MCR was slightly modified based on the previous studies (20). Chromatographic separations used an Agilent Infinity 1260 UHPLC system (Agilent, CA, United States) with a Spectrum x-aqua C18 Antiphase Column (150 × 2.1 mm, 5 μm i.d.; Beijing; China). Eluent A was 0.01% HCOOH aqueous solution (V:V) and eluent B was acetonitrile (CH_3_CN) with 0.1% formic acid (V:V). The gradient elution program was as follows: 5–60% B at 0–10 min, 60–75% at 0–20 min, 75–90% B at 20–25 min and 5% B at 25–30 min. The injection volume was 5 μL, the flow rate was 0.3 mL/min, the column temperature was 35°C, and the automatic sampler temperature was maintained at 25 ± 2°C. Diode array detector (DAD) was used to collect the data based on the full wavelength scanning method and the scan ranged from 210 to 400 nm. Mass spectrometry was performed on a Xevo G2-S QTOF mass spectrometer (Waters, Milford, MA, United States) equipped with a Dual AJD ESI (Agilent, CA, United States). The samples were analyzed in a full positive scan mode, and the scanning scope ranged from (m/z) 100 to 1,000. The parameters of mass spectrometry were as follows: dry temperature, 345°C; dry gas flow, 10 L/min; dry gas pressure, 45 psi; sheath gas temperature, 350°C; sheath gas flow, 11.0 L/min; capillary voltage, 4,000 V; cracking voltage, 135 V. Reference solutions contained purine (C5H4N4, 121.0508 amu) and HP-0921 (C18H18O6N3P3P3F24, 922.0097 amu). Data were analyzed using Agilent MassHunter Qualitative Software (Version B.05.00, Agilent Technologies, CA, United States).

### Animals, Experimental Design, and Sample Collection

The animal protocols and care standards of this experiment were accepted and approved by the Committee of Animal Care and Use of the Institute of Subtropical Agriculture, Chinese Academy of Science (Changsha, CAS20190409). Thirty-two Duroc × Large White × Landrace piglets (castrated male), weaned at an age of 21 d, were allocated randomly into four dietary treatments based on an average initial body weight of 6.37 ± 0.10 kg. Each treatment had eight replicates with one piglet each, and each replicate was assigned into an individual pen. Before starting the study, all piglets were adapted for 3 d and fed a basal diet (corn-soybean meal). Four groups included a basal diet (control, CON), the basal diet + 2,000 mg/kg MCR (LMC), the basal diet + 4,000 mg/kg MCR (MMC), and the basal diet + 8,000 mg/kg MCR (HMC). This experiment lasted for 21 d and all the piglets had unlimited access to feed and water. The formulation of basal diet met the NRC (2012) requirements for 7 ~ 11 kg growing pigs (Table 1) without antibiotics (21).

**Table 1:** Composition and calculated nutrient levels of the basal diet (air-dry basis).

**Items**	**Content**
**Ingredients (g/kg)**	
Corn	570.0
Soybean meal (43% crude protein)	220.0
Rice bran meal	50.0
Puffing maize powder	50.0
broken	50.0
Fish meal	20.0
Sucrose	10.0
Calcium lactate	3.00
Zinc oxide	2.00
Acidifier	4.00
Limestone	2.20
Monocalcium phosphate	10.0
Antioxidants	1.50
Lysine (98%)	4.00
Methionine	1.00
Threonine	1.00
Vitamin premix^a^	0.30
Mineral premix^a^	1.00
Total	1000
**Nutrient content^b^**	
Digestible energy (kcal/kg)	3274.5
Crude protein (%)	17.10
Calcium (%)	0.43
Total phosphorus (%)	0.63
Available phosphorus (%)	0.36
Lysine (%)	1.22
Methionine (%)	0.38
Methionine + cysteine (%)	0.66

a *Provided per kilogram of diet: vitamin A, 8,000 IU; vitamin D_3_, 2,000 IU; vitamin E, 300 mg; vitamin K, 30 mg; vitamin B_1_, 30 mg; vitamin B_2_, 60 mg; vitamin B_6_, 30 mg; biotin, 0.2 mg; folic acid, 10 mg; niacin, 300 mg; pantothenic acid, 300 mg; Cu ($CuSO_{\text{4}}^{.}$5H_2_O), 12 mg; Fe ($FeSO_{\text{4}}^{.}$7H_2_O), 150 mg; Mn ($MnSO_{\text{4}}^{.}$H_2_O), 5 mg; Se (NaSeO_3_), 0.45 mg; Zn (ZnO), 150 mg*.

b *Based on the composition of ingredients provided by the NRC (2012)*.

The initial and final body weights and feed intake were weighted and recorded throughout the experimental stage. The average daily weight gain (ADG), average daily feed intake (ADFI), and F/G ratio were determined. On day 22, all the piglets (8 piglets per treatment group) were stunned (250 V, 0.5 A, for 5 ~ 6 s) and killed after 12 h of fasting. Blood samples were collected from pre-caval vein and kept into vacuum tubes at room temperature for 2 h. Serum was obtained from the supernatant of blood after centrifugation at 3,500 × g for 15 min and then stored at −20°C for further analysis. Approximately 2 cm in length segments of jejunum and ileum were stored in 4% phosphate-buffered paraformaldehyde (pH 7.6) for histological analysis. Other jejunum and ileum samples flushed with 0.9% ice-cold physiological saline were immediately frozen in liquid nitrogen and stored at −80°C for molecular analysis. Colonic contents were collected for short-chain fatty acids (SCFAs) measurement; one sample of colonic content was allocated for microbiota composition determination.

### Detection of Serum Oxidative Stress Indices

The serum contents of total antioxidative capability (T-AOC), glutathione peroxidase (GSH-Px), superoxide dismutase (SOD), catalase (CAT), and malondialdehyde (MDA) were evaluated by using Spectrophotometric Kits (Nanjing Jiancheng Biotechnology Institute, Nanjing, China) according to the previous evaluation procedures (22).

### Assessment of Intestinal Morphology

The method of histological H&E staining, as previously described, was used to evaluate intestinal histomorphological changes (23). In brief, the middle sections of jejunum and ileum were embedded in paraffin after removed from fixation fluid and dehydrated, and made into ~5-μm thick transverse sections, then stained with H&E. Villus height (VH) and crypt depth (CD) were measured by using computer-assisted microscopy (Leica DMI3000B microscopy, Germany). Morphological indices were measured from 10 microscopic fields at 100× magnification. The ratio of villus height to crypt depth (VH/CD) was calculated and analyzed.

### Analysis of mRNA Expression

The extraction process of total RNA from intestinal tissues was followed by the description of Xiong et al. (24). Briefly, samples were homogenized in the Trizol Reagent (Invitrogen, Carlsbad, CA, United States) for total RNA extraction, then, further purified with the RNeasy Kit (Eppendorf AG, Hamburg, Germany). In the 10 uL reaction systems, 1.0 μg of total RNA was incubated with DNase I for synthesizing the first-strand cDNA. Then reverse transcription using Oligo (dT) primers (Takara, Otsu, Japan) was further used to synthesize the double-strand cDNA. Real-time PCR was performed with SYBR Green Master Mix reagent (Takara, Otsu, Japan) and objective gene primer pairs using the LightCycler® 480 Real-Time PCR System (Roche, Switzerland). The Primer 6.0 software was used to design primers of the β-actin housekeeping gene and target genes (Table 2). The fold changed in target genes was determined using the 2^−ΔΔCt^ method.

**Table 2:** Primers used for quantitative real-time PCR.

**Gene**	**Accession No**.	**Primer, 5′-3′**	**Size (bp)**	**T_A_ (°C)**
β-actin	XM_021086047.1	F: CTGCGGCATCCACGAAACT	147	61
		R: AGGGCCGTGATCTCCTTCTG		
IFN-γ	NM_213948.1	F: GCCATTCAAAGGAGCATGGA	144	58
		R: TTCACTGATGGCTTTGCGCT		
TNF-α	NM_214022.1	F: CCCCTGTGAGGGCAGGA	185	60
		R: CAGGCCACACATCCCTGAAT		
IL-1β	NM_214055.1	F: CCTGAGATTGATGCCGTCCA	267	60
		R: TCTTCAAGCCGTGTAGCCAT		
IL-6	NM_214399.1	F: CCTGAGATTGATGCCGTCCA	267	59
		R: TCTTCAAGCCGTGTAGCCAT		
Claudin-1	NM_001244539.1	F: AAGGACAAAACCGTGTGGGA	247	60
		R: CTCTCCCCACATTCGAGATGATT		
Occludin	NM_001163647.2	F:ACGAGCTGGAGGAAGACTGGATC	238	60
		R:CCCTTAACTTGCTTCAGTCTATTG		
ZO-1	XM_021098896.1	F: CCTGCTTCTCCAAAAACTCTT	252	60
		R: TTCTATGGAGCTCAACACCC		
IKKβ	NM_001099935.1	F: GTGACATCGCCTCTGCACTT	81	59
		R: GCAGGACGATGTTTTCTGGC		
IκBα	XM_001924394.6	F: CACCCGAGTTAGAAGGGCTC	155	59
		R: GGTATCTGCTGAGGTGTGCTG		
NF-κB	NM_001048232.1	F: AGCCATTGACGTGATCCAGG	248	60
		R: CGAAATCGTGGGGCACTTTG		

*T_A_, annealing temperature; IFN-γ, Interferon γ; TNF-α, tumor necrosis factor-α; IL-1β, interleukin-1β; IL-6, interleukin-6; ZO-1, zonula occludens-1; IKKβ, inhibiting kappa B kinase β; IκBα, inhibiting nuclear factor kappa-B; NF-κB, nuclear factor kappa-B*.

### 16S rRNA High-Through Sequencing for Microbiota Analysis

Each group selected six samples of colonic contents (*n* = 6 per group) for microbiota analysis. According to instructions of the manufacturer, total microbiota DNA was extracted using a PowerFecal^TM^ DNA isolation kit (MO BIO Laboratories, Carlsbad, CA, United States). Novogene Bioinformatics Technology Co., Ltd. was invited to complete the 16S rRNA gene sequencing. Under the PCR reaction procedure: 95°C for 30 s, 40 cycles at 95°C for 15 s, annealing at 60°C for 30 s, and at 72°C for 30 s, followed by a melting curve analysis. PCR products of the samples of the pigs were obtained using Phusion High-Fidelity PCR Mastermix [New England Biolabs (Beijing) Ltd., China], then purified by using the QIAquick Gel Extraction Kit (QIAGEN, Dusseldorf, Germany). Sequencing libraries were generated using TruSeq® DNA PCR-Free Sample Preparation Kit (Illumina, CA, United States), which subsequently enriched the recommendations and index codes of the manufacturer. The evaluation of library quality was done on the Qubit@ 2.0 Fluorometer (Thermo Fischer Scientific) and Agilent Bioanalyzer 2100 system. Finally, an Illumina MiSeq 2 × 250 platform was used to perform the library sequencing for generating 250 bp paired-end reads by the following protocols described by Caporaso et al. (25). FLASH, as a very fast and accurate analysis tool, merged paired-end reads from the original DNA fragments and then identified each sample based on the unique barcodes (26). To mine deeper data of microbial diversity of the differences between the samples, significance test were conducted with some statistical analysis methods, namely *T*-test, MetaStat, linear discriminant analysis effect size (LEfSe), Anosim, and multi-response permutation procedure (MRPP). Evaluation of the correlation between the gut microbiota and other dimensions was frequently performed with Spearman's rank correlation test. The assembled HiSeq sequences were submitted in the Sequence Read Archive (SUB8864039) of the NCBI for open access.

### Microbiological Function and Phenotypic Prediction

Based on metagenomic 16S rRNA data, Tax4Fun, as a software package, was used for predicting functional profiles (27). Tax4Fun could perform a mapping of 16 rRNA gene sequences reads to SILVA labeled OUT abundances. Normalized Taxonomic abundances are used to linearly combine the precomputed functional profiles of the KEGG organisms for predicting the microbial functional profile. BugBase is a tool for measuring high-level phenotypes in the colonic microbiota using 16S RNA datasets and mapping file (28). Besides, the Spearman correlation analysis between colonic microbiota and metabolites was performed in R software (v3.2.1).

### SCFAs Composition of Colonic Contents

The composition of SCFAs in the colonic contents was determined according to the method described by Kong et al. (29). About 1.0 g of the fresh colonic contents were mixed thoroughly with 5 mL of distilled water in a centrifuge tube, incubated for 30 min and shook, and then centrifuged at 10,000 × g, 10 min at 4°C. After transferring the supernatant into a new centrifuge tube, the precipitate was repeatedly extracted twice with 2 mL of distilled water. All supernatants (0.9 mL) were mixed with 25% metaphosphoric acid solution (0.1 mL) for 3 ~ 4 h at room temperature, then centrifuged at 10,000 × g for 10 min at 4°C. After filtration through a 0.45-μm polysulfone filter, the supernatant portion was subjected for analyses using Agilent 6890 gas chromatograph (Agilent Technologies, Inc, Palo Alto, CA, United States). The standard solutions of acetic, propionic, butyric, isobutyric, valeric, and isopentanoic acids were prepared at concentrations of 5, 10, 15, 20, and 25 mmol/L.

### Statistical Analysis

All statistical analyses were performed by using IBM SPSS 22.0 software (SPSS Inc., Chicago, IL, United States) except for the microbiome analysis. One-way ANOVA and Tukey-Kramer multiple comparison tests were used to compare the differences among the experimental treatments. Regression analysis was used to determine the linear and quadratic dose-dependent effects of MCR on the growth performance, serum antioxidant capacity, intestinal gene expression, etc. After the non-parametric tests, the 16 rRNA sequencing data were analyzed by a Kruskal–Wallis analysis to determine the significant differences. The differences were declared significant at *P* < 0.05 and a trend at 0.05 < *P* ≤ 0.10 in all analyses. The results are expressed as means ± SEM unless otherwise noted.

## Results

### Qualitative Analysis of Metabolites in the MCR

The compositions of MCR metabolites are complex and the physicochemical properties are similar, which determines the difficult separation method. As shown in Table 3, a total of 61 compounds were identified by comparing with the reference compounds and data analysis, including major compounds such as Rhodojaponia IV (peaks 7), Gallic acid (peaks 36), Epicatechin (peaks 42), Paeonolide (peaks 45), 2,4-Dimethoxybenzaldehyde (peaks 47), Albiflorin R1 (peaks 48), and Hydroxymangiferonic acid (peaks 58).

**Table 3:** Metabolites identified from the *Moutan cortex radicis*.

**Peak no**.	**Assigned identity**	**RT/min**	**Mass**	**Molecular formula**	**m/z**	**Error**	**Match score**	**Hits**
1	Gluconic acid	1.189	196.059	C6H12O7	195.051	−0.3	99.5	1
2	Cordycepic acid	1.206	192.064	C7H12O6	191.057	−0.41	86.25	3
3	Inositol-b	1.207	180.064	C6H12O6	215.033	−0.79	96.05	10
4	L-Galactoheptulose	1.209	210.075	C7H14O7	209.067	−0.78	83.59	4
5	Purine	1.212	120.043	C5H4N4	119.036	0.28	94.95	2
6	beta-Hydroxy-alpha-methylene-gamma-butyllactone	1.221	114.033	C5H6O3	113.025	−1.07	79.81	1
7	Rhodojaponia IV	1.23	454.256	C24H38O8	533.174	0.99	60.11	4
8	Ribose	1.236	150.054	C5H10O5	149.047	−1.17	92.2	4
9	Hexahydroxytaxadiene	1.245	368.219	C20H32O6	447.138	0.9	64.53	2
10	3,6-Anhydrogalactose	1.245	162.054	C6H10O5	161.047	−0.9	82.59	2
11	Andromedotoxin	1.249	412.245	C22H36O7	491.163	1.54	62.34	1
12	Manninotriose	1.253	504.171	C18H32O16	503.163	−1.56	94.79	9
13	Sarmentosin epoxide	1.255	291.096	C11H17NO8	290.089	−1	91.71	1
14	Tuliposide B	1.263	294.0979	C11H18O9	293.090	−2.81	60.17	1
15	5-O-Methylembelin	1.265	308.197	C18H28O4	387.115	2.03	56.75	1
16	Cellobiose	1.268	342.118	C12H22O11	341.110	−1.29	94.73	10
17	Allithiamine	1.29	354.118	C15H22N4O2 S2	353.110	0.73	82.47	1
18	Picrasinoside D	1.29	556.287	C28H44O11	635.206	1.76	65.38	1
19	Fumaric acid	1.318	116.012	C4H4O4	115.005	−1.16	85.31	1
20	Malic acid	1.32	134.022	C4H6O5	133.015	−0.67	84.67	1
21	Beta-D-Xylopyranosyl-(1–>6)-alpha-D-glucopyranosyl-(1–>6)-beta-D- glucopyranoside	1.334	474.159	C17H30O15	509.128	−0.11	96.33	3
22	Cepharanoline	1.346	592.263	C36H36N2O6	671.182	−5.36	40.99	3
23	Carmichaeline	1.355	377.254	C22H35N O4	456.173	2.34	52.84	3
24	Gamma-Amino-alpha-methylene butyric acid	1.369	115.064	C5H9NO2	4.057	−0.61	74.02	3
25	5alpha-Acetoxy-1 beta-benzoyl-8alpha-cinnamoyl-4alpha-hydroxy- dihydroagarofuran	1.42	562.252	C33H38O8	641.171	4.91	42.88	3
26	Linustatin	1.432	409.158	C16H27NO11	408.151	0.46	96.87	1
27	Heteratisine	1.447	391.232	C22H33NO5	470.151	3.55	42.43	1
28	Pyroglutamic acid	1.454	129.042	C5H7NO3	128.035	0.48	88.59	1
29	8-epi-Grandifloric acid	1.71	346.126	C15H22O9	345.119	0.05	84.05	4
30	Loganic acid	1.711	376.137	C16H24O10	411.106	−0.07	99.35	7
31	Yopaaoside C	1.712	422.142	C17H26O12	421.135	0	99.51	4
32	L-Arginine	2.145	174.111	C6H14N4O2	493.120	1.1	81.94	1
33	Usambarensine	2.232	432.229	C29H28N4	467.199	1.99	58.79	1
34	Ipolamiide	2.753	406.148	C17H26O11	405.140	−0.1	99.06	6
35	Maltol	2.766	126.031	C6H6O3	125.024	0.41	97.82	6
36	Gallic acid	2.769	170.021	C7H6O5	169.014	0.73	97.02	1
37	6-O-Galloyl-glucose	2.826	332.074	C13H16O10	371.067	0.03	99.85	3
38	Armillaripin	3.01	414.202	C24H30O6	493.120	2.57	54	3
39	Loganin	4.577	390.151	C17H26O10	389.143	2.07	89.34	9
40	3-Methoxygallic acid	5.872	184.037	C8H8O5	189.030	0.34	99.31	2
41	Ampelopsisin	5.879	496.158	C23H28O12	495.151	−0.36	98.93	3
42	Epicatechin	6.028	290.079	C15H14O6	289.071	0.41	99.16	9
43	p-Hydroxybenzoic acid	6.064	138.032	C7H6O3	137.024	0.02	99.89	6
44	Inumakilactone A glucoside	6.412	526.168	C24H30O13	525.161	0.7	98.87	1
45	Paeonolide	6.478	460.158	C20H28012	459.150	−0.13	98.76	3
46	Cistanoside H	6.769	506.164	C21 H30O14	505.156	−0.05	98.13	1
47	2,4-Dimethoxybenzaldehyde	7.22	166.063	C9H10O3	165.055	−0.09	98.24	8
48	Albiflorin R1	7.988	480.1633	C23H28O11	515.133	−0.13	98.2	3
49	Melampyroside	7.988	450.153	C22H26O10	449.146	−0.2	99.49	4
50	Secologanoside	8.918	556.178	C25H32O14	555.171	0.94	97.64	1
51	Inumakilactone A glucoside	9.628	526.168	C24H30O13	525.161	0.23	98.3	1
52	3,5,4'-Trihydroxystilene-4'-glucoside	10.04	390.132	C20H22O8	469.051	−0.18	47.53	10
53	N-Methyl lycodine	10.176	256.194	C17H24N2	335.113	−0.26	66.27	1
54	Benzoyl-oxypaeoniflorin	12.984	600.185	C30H32O13	599.177	−0.23	99.3	1
55	7-O-Methylaloeresin A	14.996	554.179	C29H30O11	553.171	0.24	99.65	3
56	Zizybeoside II	14.996	594.219	C25H38O16	629.188	−2.88	53.85	1
57	Benzoylpaeoniflorin	14.997	584.190	C30H32O12	619.159	−0.22	99.39	1
58	Hydroxymangiferonic acid	24.056	456.324	C29H44O4	455.317	−0.04	99.49	4
59	Queretaroic acid	25.172	472.352	C30H48O4	471.344	3.71	76.76	10
60	3-Epioleanolic acid	27.626	456.360	C30H48O3	455.353	−0.01	99.82	10
61	Chlorogenin	28.796	432.324	C27H44O4	431.317	−0.08	99.64	10

### Growth Performance

As shown in Table 4, the initial body weight of the pigs had no significant differences among the treatments (*P* > 0.05). Compared to the CON and HMC groups, the final body weight, ADG, and ADFI were significantly increased in LMC and MMC groups (*P* < 0.05). LMC significantly reduced the F/G ratio compared with the CON group (*P* < 0.05).

**Table 4:** Effects of dietary *Moutan cortex radicis* on the growth performance in growing pigs.

**Items^2^**	**Diets^1^**				***P*-value**	**Regression analysis**	
	**CON**	**LMC**	**MMC**	**HMC**		**Liner effect**	**Quadratic effect**
Initial body weight, kg	6.10 ± 0.21	6.07 ± 0.18	6.10 ± 0.18	6.11 ± 0.17	0.999	0.982	0.971
Final body weight, kg	7.93 ± 0.37^a^	11.84 ± 0.68^b^	12.51 ± 0.6^b^	8.03 ± 0.65^a^	<0.01	0.464	<0.01
ADG, g/d	80.59 ± 13.70^a^	240.72 ± 21.85^b^	282.16 ± 36.13^b^	110.48 ± 16.70^a^	<0.01	0.583	<0.01
ADFI, g/d	290.21 ± 33.52^a^	410.28 ± 26.98^b^	425.98 ± 37.06^b^	286.86 ± 37.58^a^	<0.01	0.265	<0.01
F/G ratio, g/g	3.29 ± 0.29^b^	1.72 ± 0.07^a^	2.44 ± 0.78^ab^	2.72 ± 0.47^ab^	0.039	0.630	0.019

1 *CON, control group, basal diet without antibiotics; LMC, the control diet + 2,000 mg/kg Moutan cortex radicis; MMC, the control diet + 4,000 mg/kg Moutan cortex radicis; HMC, the control diet + 8,000 mg/kg Moutan cortex radicis*.

2 *ADG, average daily gain; ADFI, average daily feed intake; F:G ratio, feed-to-gain ratio*.

*Data are expressed as means ± SEM (n = 8). Means within a row with different superscripts are significantly different (P < 0.05)*.

### Serum Antioxidant Indices

As shown in Table 5, pigs fed the MMC and HMC diets had higher T-AOC activity (*P* < 0.05) compared with the CON and LMC groups. The higher CAT activity (*P* < 0.05) was observed in the LMC and HMC pigs compared with the CON group. Compared to the MMC group, the HMC had a higher CAT activity (*P* < 0.05) in the serum. Pigs fed the CON diet had the highest GSH-Px activity (*P* < 0.05) and MDA concentration (*P* < 0.05) in the serum compared with that of the pigs fed the LMC, MMC, and HMC diets. There was no significant effect of dietary MCR on SOD activity (*P* > 0.05).

**Table 5:** Effects of dietary *Moutan cortex radicis* on serum antioxidant indexes in growing pigs.

**Items^2^**	**Diets^1^**				***P*-value**	**Regression analysis**	
	**CON**	**LMC**	**MMC**	**HMC**		**Liner effect**	**Quadratic effect**
T-AOC, U/mL	1.96 ± 0.20^a^	1.85 ± 0.08^a^	3.28 ± 0.25^b^	3.72 ± 0.26^b^	<0.01	0.682	0.671
CAT, U/mL	23.06 ± 2.45^a^	38.94 ± 3.89^bc^	32.42 ± 2.92^ab^	54.47 ± 5.06^c^	<0.01	0.834	0.927
SOD, U/mL	40.44 ± 1.88	48.94 ± 1.68	50.50 ± 4.87	52.22 ± 5.25	0.184	0.796	0.826
GSH-Px, U/mL	1238.78 ± 39.91^b^	933.63 ± 52.70^a^	946.55 ± 89.92^a^	934.80 ± 54.35^a^	<0.01	0.145	0.122
MDA, nmol/mL	10.35 ± 2.22^b^	5.11 ± 0.70^a^	2.81 ± 0.55^a^	2.53 ± 0.29^a^	<0.01	0.03	0.069

1 *CON, control group, basal diet without antibiotics; LMC, the control diet + 2,000 mg/kg Moutan cortex radicis; MMC, the control diet + 4,000 mg/kg Moutan cortex radicis; HMC, the control diet + 8,000 mg/kg Moutan cortex radicis*.

2 *T-AOC, total antioxidant capacity; CAT, catalase; SOD, superoxide dismutase; GSH-Px, glutathione peroxidase; MDA, malondialdehyde*.

*Data are expressed as means ± SEM (n = 8). Means within a row with different superscripts are significantly different (P < 0.05)*.

### Jejunal and Ileal Morphology

**Figure 1:**
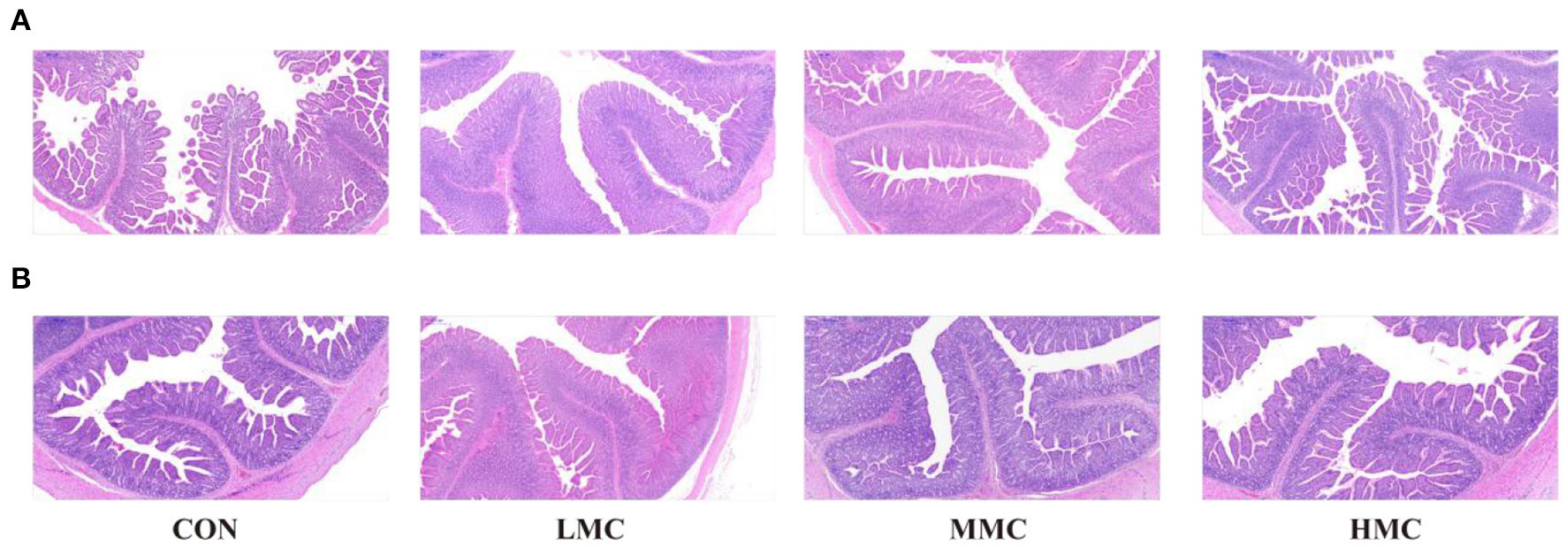
The intestinal morphology was histologically analyzed by H&E (500 μm). **(A)** Jejunum, **(B)** Ileum. CON, control group, basal diet without antibiotics; LMC, the control diet + 2,000 mg/kg *Moutan cortex radicis*; MMC, the control diet + 4,000 mg/kg *Moutan cortex radicis*; HMC, the control diet + 8,000 mg/kg *Moutan cortex radicis*.

The effects of MCR on jejunal and ileal morphology in the pigs are shown in Table 6 and Figure 1. Compared with the CON and HMC groups, the MMC significantly increased the CD (*P* < 0.05) in the jejunum. The LMC diet increased the ratio of VH/CD (*P* < 0.05) in the jejunum compared with the MMC diet. Pigs fed with the HMC diet had the shortest VH (*P* < 0.05) compared with that of the pigs fed the other three diets. Compared to the CON and HMC diets, LMC and MMC diets markedly increased (*P* < 0.05) the VH and CD in the ileum.

**Table 6:** Effects of dietary *Moutan cortex radicis* on the morphology of the jejunum and ileum of growing pigs.

**Items**	**Diets^1^**				***P*-value**	**Regression analysis**	
	**CON**	**LMC**	**MMC**	**HMC**		**Liner effect**	**Quadratic effect**
**Jejunum**							
Villus height, μm	370.13 ± 9.21^b^	382.23 ± 9.92^b^	372.27 ± 8.58^b^	340.50 ± 7.89^a^	<0.01	0.013	<0.01
Crypt depth, μm	200.39 ± 7.01^b^	189.56 ± 6.53^ab^	225.77 ± 6.44^c^	175.80 ± 5.47^a^	<0.01	0.025	<0.01
Villus height/Crypt depth	2.00 ± 0.09^ab^	2.26 ± 0.11^b^	1.7 ± 0.08^a^	2.06 ± 0.08^ab^	<0.01	0.957	0.829
**Ileum**							
Villus height, μm	312.28 ± 8.34^a^	358.50 ± 5.29^b^	343.82 ± 14.67^b^	323.20 ± 7.46^a^	<0.01	0.065	<0.01
Crypt depth, μm	174.49 ± 6.20^a^	212.31 ± 4.63^b^	206.47 ± 5.68^b^	185.63 ± 5.19^a^	<0.01	0.174	<0.01
Villus height/Crypt depth	1.77 ± 0.05	1.78 ± 0.04	1.82 ± 0.04	1.84 ± 0.05	0.746	0.648	0.410

1 *CON, control group, basal diet without antibiotics; LMC, the control diet + 2,000 mg/kg Moutan cortex radicis; MMC, the control diet + 4,000 mg/kg Moutan cortex radicis; HMC, the control diet + 8,000 mg/kg Moutan cortex radicis*.

*Data are expressed as means ± SEM (n = 8). Means within a row with different superscripts are significantly different (P < 0.05)*.

### Expression of Genes Associated With Pro-Inflammatory Factors, Tight Junction Proteins, and NF-κb Signaling Pathway

**Figure 2:**
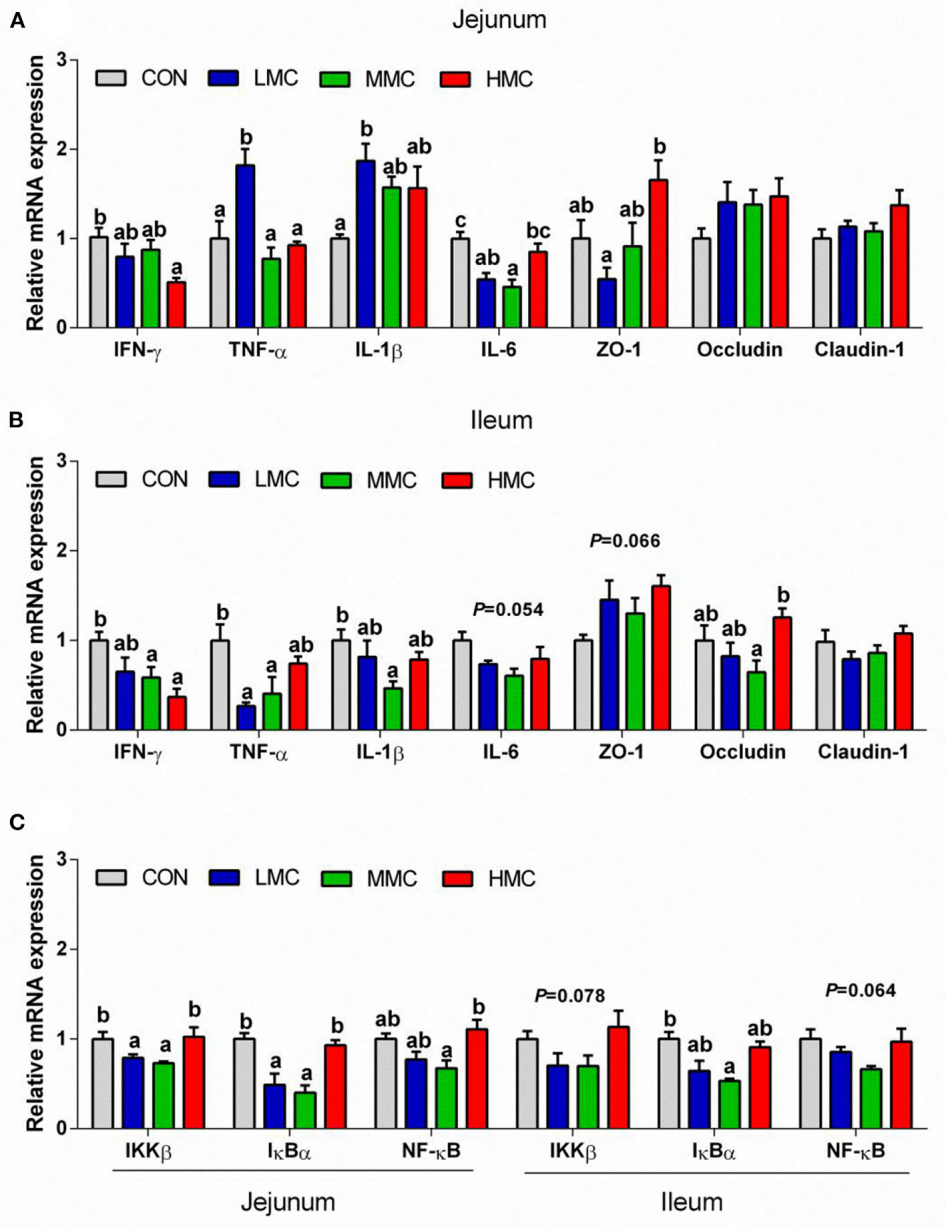
Gene expression levels associated with the proinflammatory factors (IFN-γ, TNF-α, IL-1ß, and IL-6) and tight junction proteins (ZO-1, occludin, and claudin-1) of jejunum **(A)** and ileum **(B)**, and NF-κB signaling pathway in the jejunum and ileum tissues **(C)** of pigs fed the *Moutan cortex radicis* diet. CON, control group, basal diet without antibiotics; LMC, the control diet + 2,000 mg/kg *Moutan cortex radicis*; MMC, the control diet + 4,000 mg/kg *Moutan cortex radicis*; HMC, the control diet + 8,000 mg/kg *Moutan cortex radicis*. Data are expressed as means ± SEM (*n* = 6). Means with different superscripts in the columns are significantly different (*P* < 0.05).

As shown in Figure 2, the HMC group significantly decreased the mRNA expression level of interferon γ (IFN-γ, *P* < 0.05) in the jejunum and ileum compared with the CON group. Compared to the CON diet, the MMC diet decreased the mRNA expression level of interleukin-6 (IL-6, *P* < 0.05) in the jejunum and mRNA expression levels of tumor necrosis factor-α (TNF-α) and IL-1β in the ileum (*P* < 0.05). The LMC diet significantly inhibited the mRNA expression level of TNF-α (*P* < 0.05) in the ileum compared with the CON diet but had the highest mRNA expression level of TNF-α (*P* < 0.05) in the jejunum of pigs among all the treatments. Dietary supplementation of MCR had a trend of an inhibited IL-6 mRNA expression (*P* = 0.054). The HMC significantly enhanced (*P* < 0.05) the ZO-1 mRNA expression in the jejunum compared with the LMC diet and increased (*P* < 0.05) the occludin mRNA expression in the ileum compared to the MMC diet. And there was a trend (*P* = 0.066) toward a higher mRNA expression level of ZO-1 in the ileum pigs fed MCR diets.

The influences of dietary MCR on the IKKβ/IκBα/NF-κB signaling pathway are shown in Figure 2C. Compared to the CON and HMC diets, the LMC and MMC diets significantly inhibited (*P* < 0.05) the expressions of inhibiting kappa-B kinase β (IKKβ) and inhibiting nuclear factor kappa-B (IκBα) in the jejunum. The MMC group had a lower (*P* < 0.05) expression level of NF-κB mRNA in the jejunum compared with the HMC group. The LMC and MMC groups had a decreasing tendency for the mRNA expression levels of IKKβ (*P* = 0.078) and NF-κB (*P* = 0.064) in the ileum compared with the CON and HMC groups. Compared to the CON group, the MMC group also significantly downregulated the IκBα mRNA expression (*P* < 0.05) in the ileum.

### Concentrations of SCFA in the Colonic Contents

**Figure 3:**
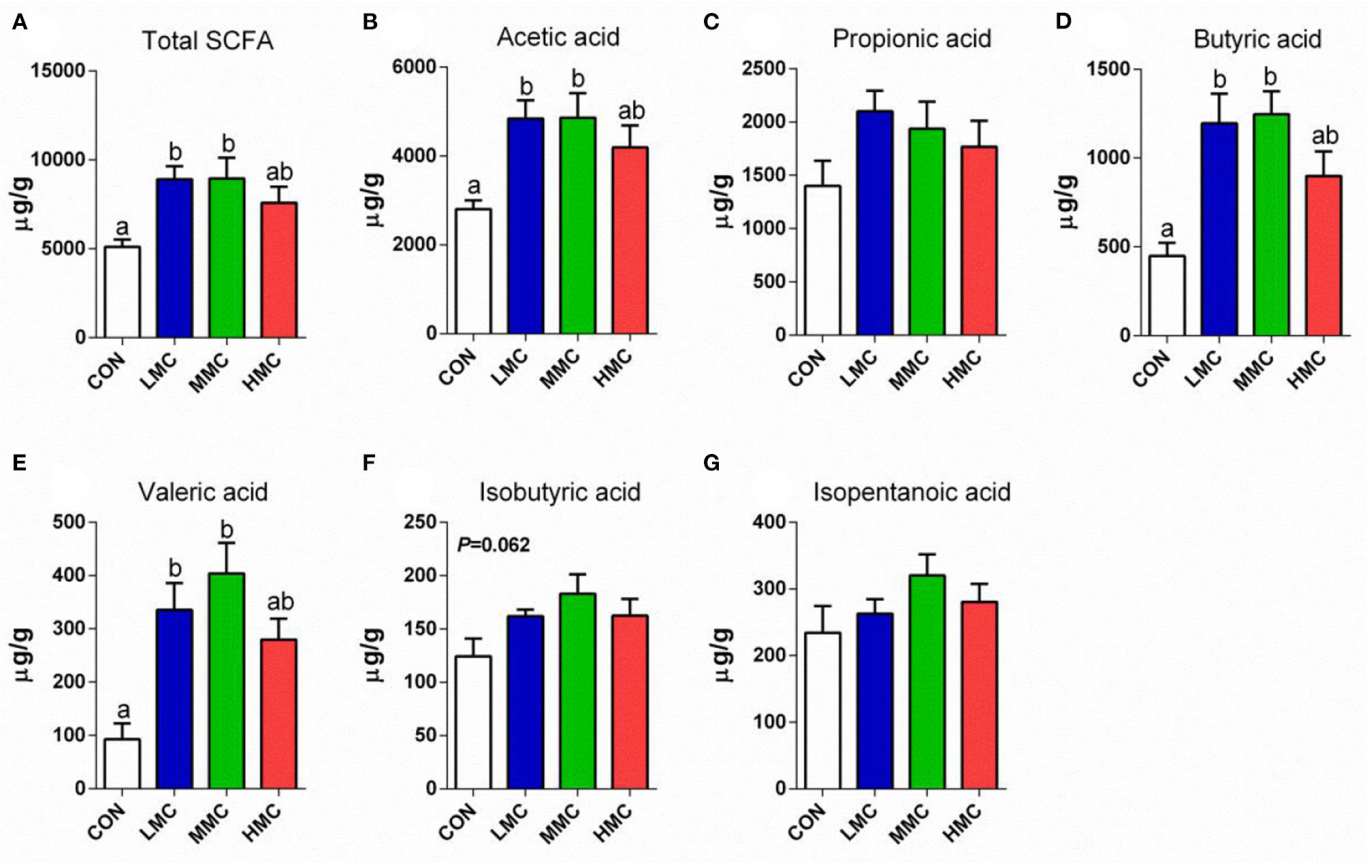
Total SCFAs **(A)**, acetic acid **(B)**, propionic acid **(C)**, butyric acid **(D)**, valeric acid **(E)**, isobutyric acid **(F)**, and isopentanoic acid **(G)** concentrations in colonic contents of pigs fed the *Moutan cortex radicis* diet for 3 weeks. CON, control group, basal diet without antibiotics; LMC, the control diet + 2,000 mg/kg *Moutan cortex radicis*; MMC, the control diet + 4,000 mg/kg *Moutan cortex radicis*; HMC, the control diet + 8,000 mg/kg *Moutan cortex radicis*. Data are expressed as means ± SEM (*n* = 8). Means with different superscripts in the columns are significantly different (*P* < 0.05).

Analysis of the concentrations of SCFA in the colonic contents revealed differences among all the treatments (Figure 3). The concentrations of total SCFA, acetic acid, butyric acid, and valeric acid in the colonic contents were higher in the LMC and MMC groups than in the CON group (*P* < 0.05). Dietary supplementation of MCR showed a tendency to increase the concentration of isobutyric acid (*P* = 0.062) in the colonic contents of pigs. The concentrations of propionic acid and isopentanoic acid among the four treatments had no significant difference (*P* > 0.05).

### Colonic Microbiota Diversity and Composition

**Figure 4:**
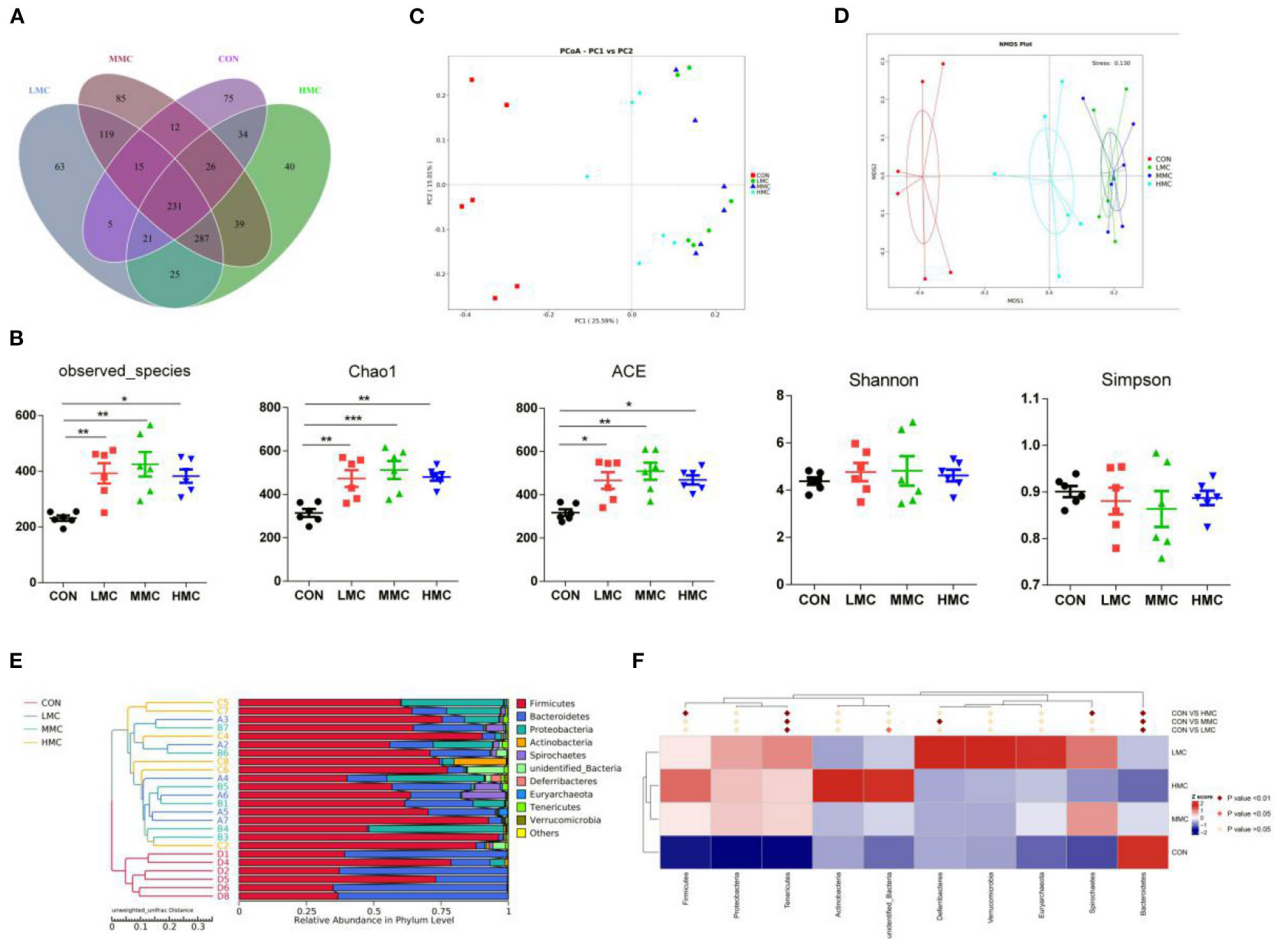
Effect of dietary *Moutan cortex radicis* on the colonic microbiota diversity and composition in the pigs. **(A)** A Venn diagram illustrating the overlaps of OTUs in the gut microbiota; **(B)** The microbial alpha diversity indices (Observed-species, Chao1, Shannon, Simpson, ACE) were calculated using the mothur program; **(C)** Principal coordinate analysis (PCoA); **(D)** non-metric multidimensional scaling (NMDS) analysis; **(E)** unweighted UniFrac cluster tree based on Unweighted Pair-group Method with Arithmetic Mean (UPGMA) analysis; **(F)** The significant different species among groups based on MetaStat analysis. CON, control group, basal diet without antibiotics; LMC, the control diet + 2,000 mg/kg *Moutan cortex radicis*; MMC, the control diet + 4,000 mg/kg *Moutan cortex radicis*; HMC, the control diet + 8,000 mg/kg *Moutan cortex radicis*. Data are expressed as means ± SEM (*n* = 6). *P < 0.05, **P < 0.01, and ***P < 0.001.

To better understand the differences in richness, the overlaps among treatments were illustrated using a Venn diagram (Figure 4A). This analysis showed that CON and LMC, CON and MMC, and CON and HMC contained 304, 319, and 361 common operational taxonomic units (OTUs), respectively. As shown in Figure 4B, the microbial richness indices (Chao1, ACE, and observed species) were significantly increased (*P* < 0.05) in the gut microbiota of the piglets with MCR supplementation, whereas no significant differences were found in the diversity indices (Shannon and Simpson) of gut microbiota. The principal coordinate analysis (PCoA, Figure 4C) and non-metric multidimensional scaling (NMDS, Figure 4D) analysis of β-diversity showed a strong difference in the microbiota from the control group to MCR-treated groups. An unweighted UniFrac cluster tree based on the unweighted pair-group method with arithmetic mean (UPGMA) analysis showed the similarity and phylogeny of all observed samples at the phylum level (Figure 4E) and *Firmicutes, Bacteroidetes*, and *Proteobacteria* are the dominant bacteria in the colonic microbiota of the pigs. Further, MetaStat analysis of the microbial community was to explore the significant differences in microbial composition between the MCR-treated group and the control group (Figure 4F). MCR supplementation significantly elevated the relative abundance of *Tenericutes* and decreased the relative abundance of *Bacteroidetes* in the colonic microbiota.

**Figure 5:**
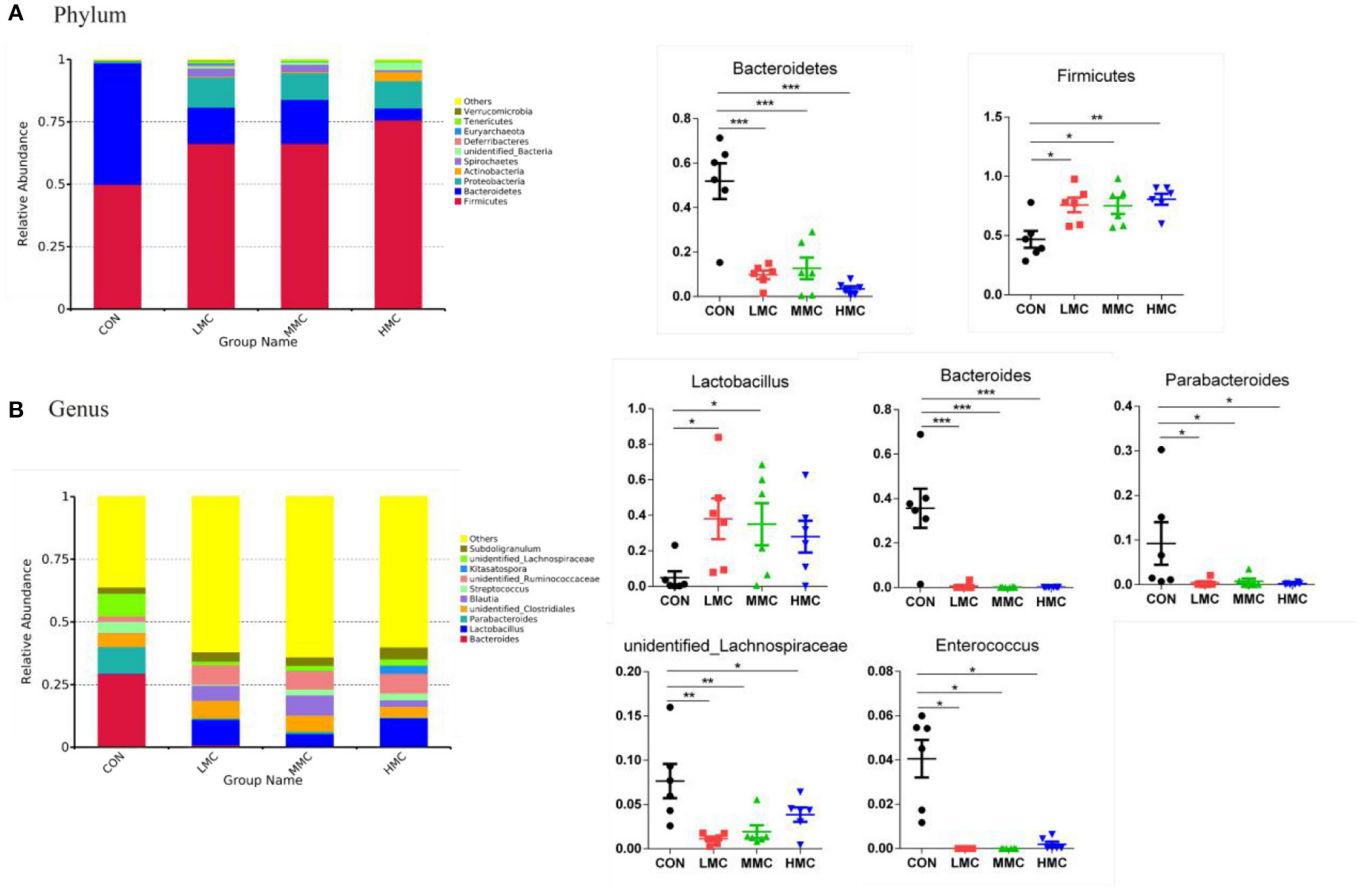
Effects of dietary *Moutan cortex radicis* on microbial composition at the phylum and genus levels of growing pigs. **(A)** Relative contribution of the top 10 phylum in each group (left) and the relative abundance of significantly different microorganisms (right); **(B)** Relative contribution of the top 10 genus in each group (left) and the relative abundance of significantly different microorganisms (right). CON, control group, basal diet without antibiotics; LMC, the control diet + 2,000 mg/kg *Moutan cortex radicis*; MMC, the control diet + 4,000 mg/kg *Moutan cortex radicis*; HMC, the control diet + 8,000 mg/kg *Moutan cortex radicis*. Data are expressed as means ± SEM (*n* = 6). *P < 0.05, **P < 0.01, and ***P < 0.001.

As shown in Figure 5, the phylum level analysis showed that dietary supplementation of MCR significantly increased the relative abundance of *Firmicutes* (*P* < 0.05) and decreased the relative abundance of *Bacteroidetes* (*P* < 0.05). In the genus level, MCR treatment significantly decreased the relative abundances of *Bacteroides, Parabacteroides, unidentified_Lachnospiraceae*, and *Enterococcus* in the colonic microbiota (*P* < 0.05). Compared to the CON group, LMC and MMC groups increased (*P* < 0.05) the relative abundance of *Lactobacillus*.

### Metabolic Functions and Phenotypes of Colonic Microbiota

**Figure 6:**
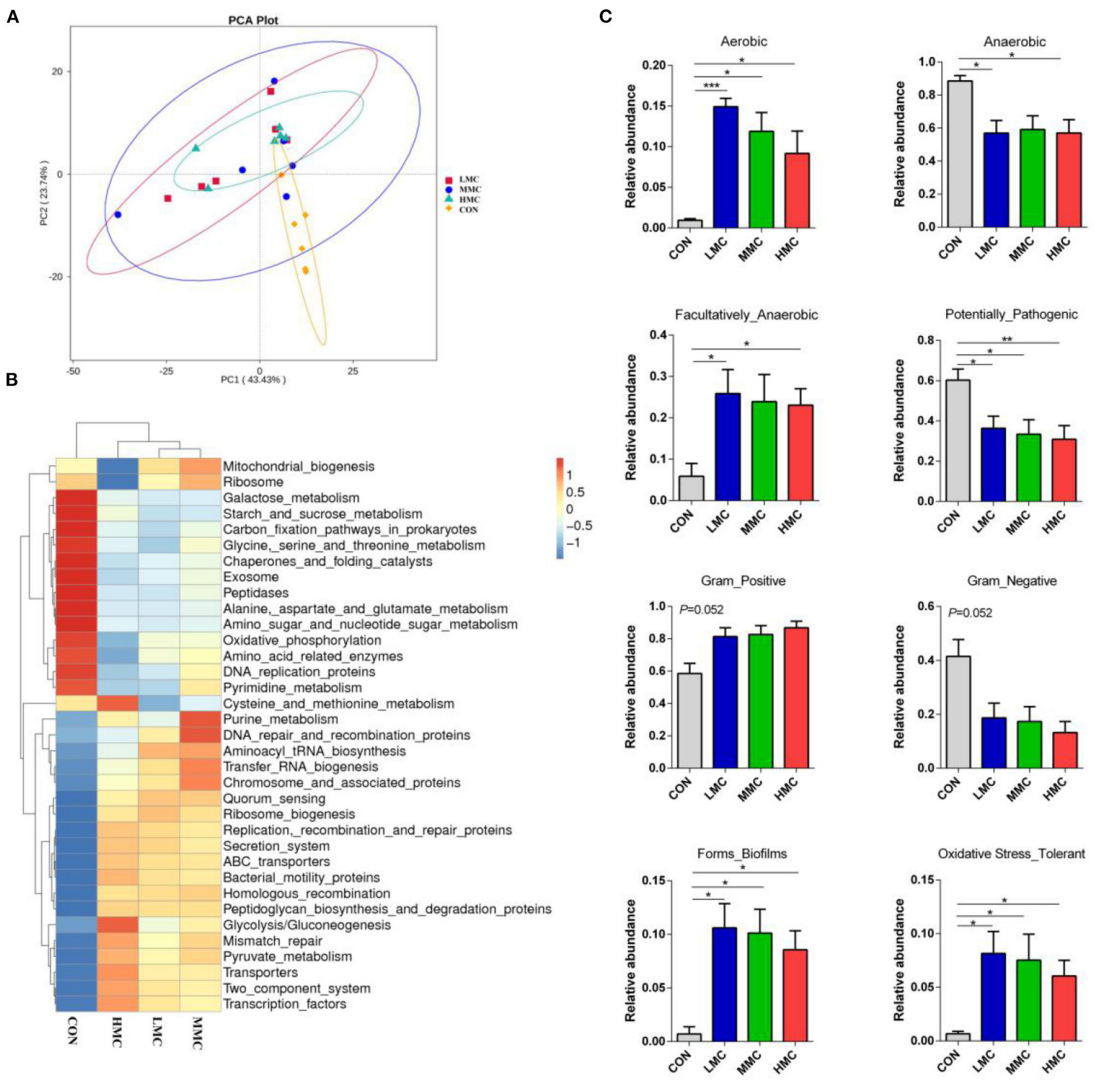
Dietary supplementation of *Moutan cortex radicis* altered the metabolic functions and phenotypes of colonic microbiota in the pigs. **(A)** Principal components analysis (PCA) of functional profiles in the gut microbiota; **(B)** The heatmap tree based on different metabolism-related pathways at KEGG level 3; **(C)** The metabolic phenotypes prediction were compared using BugBase online (https://bugbase.cs.umn.edu/). The relative abundances of discrete phenotype were performed using pair-wise Mann-Whitney *U*-tests. Data are expressed as means ± SEM (*n* = 6). CON, control group, basal diet without antibiotics; LMC, the control diet + 2,000 mg/kg *Moutan cortex radicis*; MMC, the control diet + 4,000 mg/kg *Moutan cortex radicis*; HMC, the control diet + 8,000 mg/kg *Moutan cortex radicis*. *P < 0.05, **P < 0.01, and ***P < 0.001.

Tax4Fun was performed to determine the effects on metabolic functions of gut microbiota by MCR treatment. Based on the KEGG annotation results, the principal components analysis (PCA) showed that the microbiotal metabolic functions were significantly separated in the CON groups and MCR-treated groups (Figure 6A). As shown in Figure 6B, KEGG pathways associated with microbial metabolism at level 3, including mismatch repair, pyruvate and purine metabolism, DNA repair, and recombination protein were upregulated by dietary MCR-treated. Galactose metabolism, oxidative phosphorylation, and amino acid-related enzymes were significantly downregulated. Moreover, based on 16S OTU results to predict bacterial phenotype database, BugBase can analyze the differences among the groups simultaneously. As Shown in Figure 6C, the MCR diet significantly increased (*P* < 0.05) the aerobic bacterial richness and oxidative stress tolerance and biofilm forming of colonic microbiota compared to the CON group. The richness of Gram-positive bacteria showed a marked increasing trend (*P* = 0.052), while Gram-negative bacteria had a decreasing trend (*P* = 0.052) by MCR treatments. The pathogenic potential of the gut microbiota was reduced (*P* < 0.05) by increasing the dietary MCR level. The LMC and HMC groups had a lower (*P* < 0.05) richness of anaerobic bacteria and a higher (*P* < 0.05) facultative anaerobic bacteria than the CON group.

**Figure 7:**
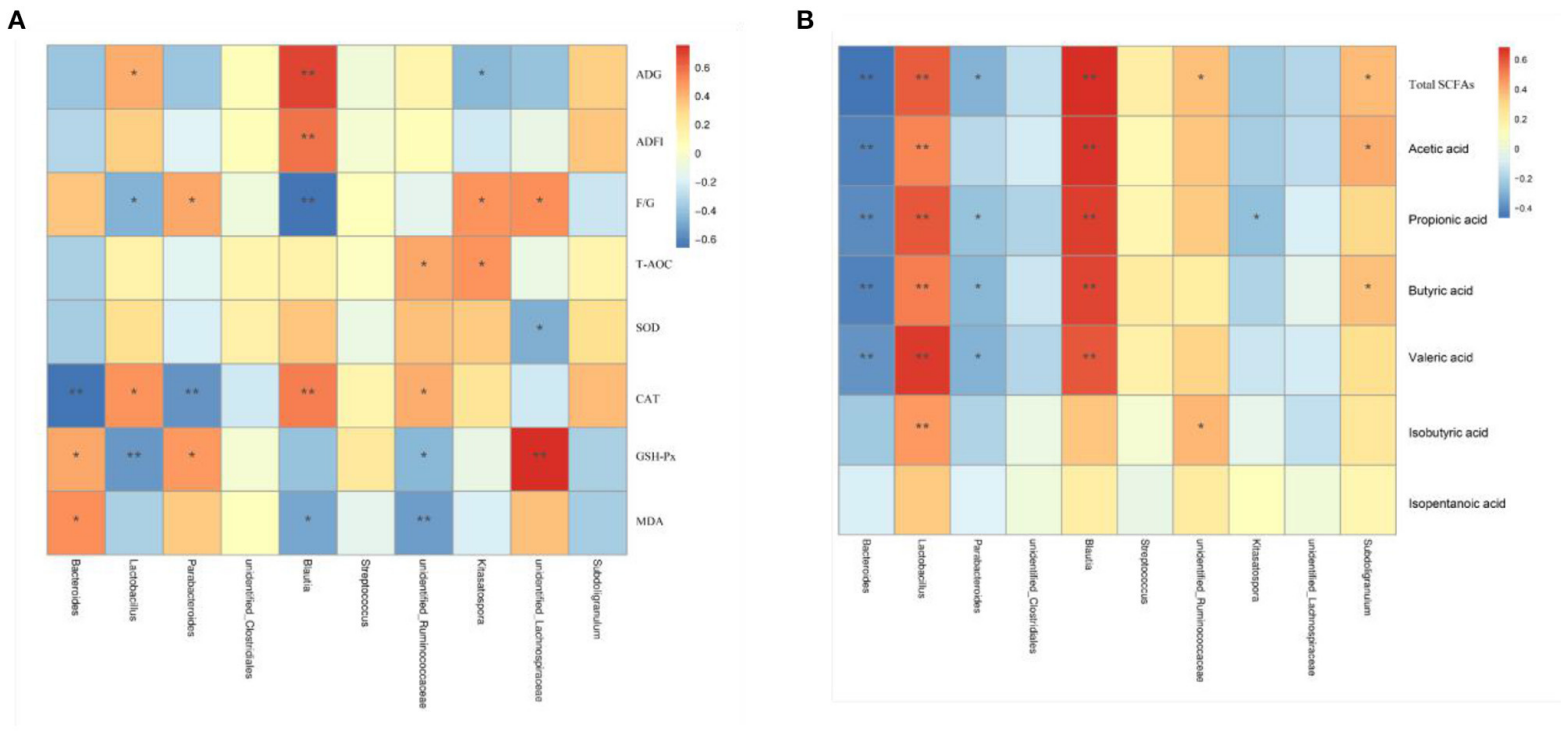
Heatmap of the Spearman's r correlations analysis. Spearman's correlation coefficients between colonic microbiota and growth and serum antioxidant parameters **(A)**, and colonic SCFA contents **(B)**. *P < 0.05 and **P < 0.01.

Results of Spearman's correlation coefficients between the major genera and growth, serum antioxidant parameters makers and colonic SCFA contents were calculated and presented with heatmap (Figure 7). *Lactobacillus* and *Blautia* had significant positive relations with ADG, serum CAT activity, and the contents of total SCFAs, acetic acid, propionic acid, butyric acid, and valeric acid (*P* < 0.05) and was negatively related with the F/G ratio (*P* < 0.05). *Bacteroides* showed significant positive relations with serum GSH-Px activity and MDA content and was negatively correlated with CAT and the contents of total SCFAs, acetic acid, propionic acid, butyric acid, and valeric acid (*P* < 0.05). *Parabacteroides* showed significant positive correlations with serum GSH-Px activity and the F/G ratio (*P* < 0.05) and was negatively correlated with CAT and total SCFAs, propionic acid, butyric acid, and valeric acid contents (*P* < 0.05). *Unidentified_Lachnospiraceae* was positively correlated with (*P* < 0.05) the F/G ratio and GSH-Px activity and negatively correlated with (*P* < 0.05) serum SOD activity.

### Regression Analysis

As shown in Table 7, the linear and quadratic dose-dependent effects of MCR on the determinations of growth performance, serum antioxidant capacity, and intestinal gene expression were analyzed by the regression analysis. The regression equations were established for calculating the optimal additive dose of MCR according to the significant differences (*P* < 0.05 and *R*^2^ > 0.40). Final body weight, ADG and ADFI, and the VH and CD of ileum were quadratic negative correlated with the MCR level (*P* < 0.05 and *R*^2^ > 0.40). The F/G ratio, the VH and CD, and the relative expression of IL-6, NF-κB, IκBα, and IKKβ mRNA of jejunum, the ratio of VH/CD and the relative expression of IKKβ mRNA of ileum had a quadratic positive correlation with dietary MCR level (*P* < 0.05 and *R*^2^ > 0.40). There was a linear negative correlation between the serum MDA content and the MCR level (*P* < 0.05), indicating that MDA content decreased with the increase of MCR supplemental level. The optimal MCR supplemental level for growth performance, serum antioxidant capacity, and intestinal health of weaned piglets was 3,420 ~ 4,237 mg/kg.

**Table 7:** Regression analysis.

**Items (Y)**	**Correlation**	**Optimum addition amount of *Moutan cortex radicis* (X, mg/kg)**
Final body weight, kg	Y = −2.853E-07X^2^ + 0.002X + 7.82	4,029
ADG, kg/d	Y = −1.11E-08X^2^ + 8.96E-05X + 0.08	4,044
ADFI, kg/d	Y = −8.450E-09X^2^ + 6.497E-05X + 0.299	3,844
F/G ratio	Y = 1.497E-07X^2^-0.001X + 4.816	4,579
MDA, nmol/mL	Y = −0.026X + 224.251	
**Jejunum**		
Villus height, μm	Y = 3.565E-05X^2^-0.290X + 893.078	4,063
Crypt depth, μm	Y = 1.768E-05X^2^-0.144X + 472.728	4,085
IL-6 mRNA expression	Y = 3.11E-08X^2^ + 0.000X + 0.986	4,237
NF-κB mRNA expression	Y = 2.314E-08X^2^ + 0.000X + 1.006	3,732
IκBα mRNA expression	Y = 3.688E-08X^2^ + 0.000X + 0.984	4,083
IKKβ mRNA expression	Y = 1.809E-08X^2^ + 0.000X + 0.999	3,875
**Ileum**		
Villus height, μm	Y = −5.445E-06X^2^ + 0.044X + 790.553	4,053
Crypt depth, μm	Y = −4.853E-06X^2^ + 0.041X + 446.152	4,191
Villus height/Crypt depth ratio	Y = 1.081E-08X^2^ + 0.000X + 1.1996	5,042
IKKβ mRNA expression	Y = 2.691E-08X^2^ + 0.000X + 0.987	3,420

## Discussion

In recent years, the misuse of feed antibiotics in the swine industry has seriously threatened human health and food safety, and China has banned the application of antibiotics in feeds in 2020. Therefore, exploring an alternative to antibiotics is necessary for the sustainable development of the livestock industry. Many previous studies have found the positive results of MCR in various animal models of disease, such as mouse, rat, and human (30–32). In the present study, the addition of MCR to the diet without antibiotics showed the effect of promoting growth performance in weaned piglets. The improvement may be due to protecting piglets from oxidative stress and intestinal inflammation response caused by weaning stress, which was evidenced by the enhanced antioxidant capacity, inhibition of NF-κB signaling pathway, and regulation of intestinal microbiota structure and metabolites in piglets.

The depletion of intracellular free radicals and antioxidants inhibited various antioxidant enzyme activities, which induced oxidative stress (33). The antioxidant mechanism of polyphenols mainly through increasing antioxidant protective barrier and eliminating intracellular ROS to maintain oxidative balance (34, 35). Previous studies demonstrated that more than 50 μg/mL of MCR enhanced the antioxidant defense system by improving the activities of GSH and SOD in glucose-induced oxidative damage (36). In this study, the activities of T-AOC and CAT were improved and the GSH-Px activity was decreased in weaned piglets supplemented with MCR. Overall, MCR can play an antioxidant role by increasing antioxidant activity. The mechanism of anti-oxidative stress and anti-inflammation closely connected to the NF-κB signaling pathway in the body (37, 38). Dynamic changes of pro-inflammatory cytokines levels in the intestinal tract tissue act as crucial messengers to stimulate the intestinal inflammatory process. Therefore, during anti-inflammatory therapy, it is necessary to downregulate the production of these pro-inflammatory cytokines (39). The phosphorylation and degradation of the NF-κB-bound protein IκB, activated by the IKKβ signaling phosphorylation, are directly involved in the activation of NF-κB (40). As demonstrated in the present study, MCR has effectively decreased the cytokine productions in jejunum and ileum *via* inhibiting the IKKβ/IκBα/NF-κB signaling pathway. At the same time, evidence was also found for the fact that MCR or paeonol could suppress the gene and protein expression of pro-inflammatory cytokines by blocking the NF-κB pathway in the LPS-stimulated inflammatory response (31, 41). Thus, it could be suggested that MCR has potential in antioxidant and anti-inflammation therapy in weaned piglets.

Enhanced intestinal morphology and gut barrier are closely associated with nutrients absorption and intestinal integrity (42). Intestinal morphology significantly changes, including villous atrophy and crypt hyperplasia, which will result in diarrhea and growth retardation in pigs (43). An increasing VH/CD ratio is one of the most important indices of intestinal morphology in evaluating the improvement of intestinal function and enhancement of absorption capacity (44). A recent study found that the dietary supplemented with MCR at 2,000 mg/kg improved the ratio of VH to CD in the jejunum and increased the VH and CD in the ileum of weaned piglets. It was found that 4,000 mg/kg MCR increased the VH and CD in the ileum, whereas 8,000 mg/kg MCR decreased the VH and CD in the jejunum and ileum compared with 2,000 and 4,000 mg/kg MCR groups. Therefore, we speculated that a high dosage (8,000 mg/kg) of MCR may be harmful to the improvement of intestinal villi and intestinal digestive ability. Tight junctions protein, as the mechanical barrier, constitutes intestinal barrier function and prevents pathogenic antigen invasion (45). Occludin, claudin-1, and ZO-1 are the main cytoplasmic transmembrane and adaptor protein and jointly constitute the tight intercellular junctions. Improved expression of three crucial proteins can enhance the intestinal barrier function for decreasing permeability of the intestinal wall (46). Several studies have found that traditional Chinese medicine can alter intestinal permeability dependent on tight junction protein changes (47, 48). The results also demonstrated that ZO-1 and occludin mRNA expression in jejunum and ileum were increased in piglets fed MCR (8,000 mg/kg feed) diet. This suggests that a high dosage of MCR contributed to improving the intestinal barrier integrity in weaned piglets.

The gut microbiome is a complex microbial ecosystem, whose activities and reciprocal relationship have been essential to the host health and disease (49). The investigation of the gut microbiome has been described as a biomarker for evaluating the effect of specific dietary components on the host. In the current research, MCR shapes intestinal microbiota in weaned piglets, including increases in the microbial richness; the abundances of the phyla *Firmicutes* and the genera *Lactobacillus*; and a decrease in the abundances of the phyla *Bacteroidetes* and the genera *Bacteroides, Parabacteroides, unidentified_Lachnospiraceae*, and *Enterococcus*. Piglets fed MCR diets had a higher observed Chao1, ACE, and species number for gut microbiota, which indicates that MCR supplementation contributes to improving microbial diversity. *Firmicutes* and *Bacteroidetes*, as two main communities, are associated with energy metabolism homeostasis (50). Many previous studies reported that increased *Firmicutes* and reduced *Bacteroidetes* are most common in the obesity phenotype, which led to effective absorption of the calories from food (51). Therefore, MCR could increase the growth performance of the piglets that may be closely related to the variation of the gut microbiota composition. The abundance of *Lactobacillus* in the intestine is closely related to activating the production of secretory IgA for improving intestinal mucosal immunity, which acts an important role in maintaining intestinal barrier function (52). *Bacteroides* and *Parabacteroides*, occurring in the early stages of life, have been reported to produce gamma amino butyric acid, associated with growth (53). An increase in the abundance of *Bacteroides* is usually found in the occurrences of ulcerative colitis, colorectal cancer, and functional gastrointestinal disorders (54). The abundance of *Enterococcus* correlated positively with metabolites associated with inducing oxidative stress (55). Moreover, changed microbial composition has been linked to the production and composition of SCFA in the colon. In the present study, we found that colonic contents of SCFA, including acetic acid, propionic acid, butyric acid, and valeric acid, were increased significantly in piglets fed the MCR diet at 2,000 and 4,000 mg/kg. SCFA, as an important metabolite of gut microbiota, could favor energy homeostasis and relieve inflammations and metabolic syndrome in the colon (56). Corrêa-Oliveira has demonstrated that the addition of SCFA increased villi height and crypt depth, enhanced the intestinal barrier, and had anti-inflammatory properties in mice (57). In summary, MCR addition regulates the intestinal microbiota and microbial metabolites of the piglets for improving intestinal health. And it would be interesting to further investigate whether MCR has a marked influence on lipid metabolism through regulating intestinal microbiota in weaned piglets.

Based on microbial function prediction, results demonstrated that the MCR addition increased the pyruvate metabolism, DNA repair, and purine metabolism, and decreased oxidative phosphorylation and amino acid-related enzymes. MCR may inhibit the amino acid metabolism and promote nucleotide metabolism and multi-drug resistance in gut microbial communities. Moreover, the changes of microbial metabolic phenotypes in weaned piglets treated with different doses of MCR were first revealed. Dietary supplementation of MCR has a strong antimicrobial property against Gram-negative and anaerobic bacteria but promotes the proliferation of Gram-positive and aerobic bacteria. MCR supplementation also increased biofilm forming and oxidative stress tolerance, while the promoting effect was negatively correlated with the added dose. Biofilm formation and oxidative stress tolerance of microbial communities were found to go together with drug resistance, inflammation, and pathogenesis (58). Higher MCR levels significantly reduced the pathogenic potential of microbial communities. However, these metabolic phenotypes changes need to further explore the mechanism. Further, association analysis of growth performance, serum antioxidants, colonic SCFA contents, and microbiota first revealed that MCR supplementation has widely influenced the growth and health of piglets.

## Conclusion

In conclusion, dietary supplemented with MCR was able to significantly alleviate weaning stress in piglets, as demonstrated by improving antioxidant capacity and regulating gut microbial communities. MCR increased serum antioxidant capacity, improved intestinal barrier function, and inhibited the NF-κB signaling pathway. Additionally, besides improving the richness indices, MCR significantly increased the microbial metabolic phenotypes and functions, and metabolites, which benefit weaned piglets with better intestinal status and growth potential. The present study contributes to providing theoretical support in applying MCR at 3,420 ~ 4,237 mg/kg for antioxidation and regulating intestinal health in livestock production.

## Data Availability Statement

The datasets presented in this study can be found in online repositories. The names of the repository/repositories and accession number(s) can be found at: NCBI SRA; PRJNA690218, Figshare; doi: 10.6084/m9.figshare.14502873, https://figshare.com/s/3dd56c3a72e0153a8ee7.

## Ethics Statement

The animal study was reviewed and approved by the Committee of Animal Care and Use of the Institute of Subtropical Agriculture, Chinese Academy of Science (Changsha, CAS20190409). Written informed consent was obtained from the owners for the participation of their animals in this study.

## Author Contributions

MB conducted the animal work, sample analysis, and manuscript writing. HL, JD, YY, and QS designed the research and reviewed the manuscript. KX, XX, and RH analyzed the data and helped revise the manuscript. ZL and QS provided experimental materials and analyzed study data. SW and JZ helped conduct animal trials and sample analysis. All authors read and approved the final manuscript.

## Conflict of Interest

The authors declare that the research was conducted in the absence of any commercial or financial relationships that could be construed as a potential conflict of interest.
